# Regulatory T cell induction by mesenchymal stem cells depends on the expression of TNFR2 by T cells

**DOI:** 10.1186/s13287-020-02057-z

**Published:** 2020-12-10

**Authors:** Sina Naserian, Sara Shamdani, Nassim Arouche, Georges Uzan

**Affiliations:** 1 INSERM UMR-S-MD 1197, Hôpital Paul Brousse, Villejuif, France; 2 Paris-Saclay University, Villejuif, France; 3 CellMedEx, Saint Maur Des Fossés, France

**Keywords:** Mesenchymal stem cells, Regulatory T cells, TNF-TNFR2 signaling pathway, Immune checkpoint, Immunosuppression, Immunoregulation, Immune therapy

## Abstract

Mesenchymal stem/stromal cells can modulate the effector immune cells especially T lymphocytes. Due to this important feature, they can regulate the development of a variety of disorders including inflammatory and autoimmune disorders, cancers, and transplantation outcomes. One of the most important MSC immunoregulatory functions is their capacity to convert conventional T cells into regulatory T cells. Several mechanisms, mostly related to MSCs but not T cells, have been shown essential for this aspect. The inflammatory microenvironment majorly caused by pro-inflammatory cytokines has been demonstrated to govern the direction of the immune response. In this respect, we have recently revealed that the TNFα-TNFR2 signaling controls several aspects of MSC immunomodulatory properties including their ability to suppress T cells and their conversion towards Foxp3-expressing Tregs. Here in this work, we have looked from another angle by investigating the impact of TNFR2 expression by T cells on their ability to be converted to suppressive Tregs by MSCs. We showed that unlike WT-T cells, their TNFR2 KO counterparts are remarkably less able to convert into Foxp3^+^ and Foxp3^−^ Tregs. Furthermore, TNFR2 blockade diminished the anti-inflammatory cytokine secretion by iTregs and consequently resulted in less T cell immunosuppression. This work is the first evidence of the crucial association of TNFR2 expression by T cells with their iTreg conversion capacity by MSCs. It strengthens once more the potential of anti-TNFR2 administration for a strong and effective interference with the immunosuppression exerted by TNFR2-expressing cells.

## Background

Mesenchymal stem/stromal cells (MSCs) are multipotent stem cells with unique biological potentials. Among them, their regenerative and immunoregulatory functions are the most important, making them the ideal choices for cell therapy applications. MSCs are able to modulate immune cells especially T lymphocytes. They can suppress conventional T cells (Tconvs) and convert them to regulatory T cells (Tregs) [1]. Indeed, we and others have reported several mechanisms behind their capacity to induce Tregs. The modulation of ubiquitination factors [2], Treg-specific *demethylated* region (TSDR) demethylation [2], microRNAs such as miR126a [3], and runt-related transcription factor (RUNX) complex are some of the principal mechanisms [4]. Pro-inflammatory environment plays a crucial role in the MSC immunoregulatory function. For instance, pre-treating MSCs with tumor necrosis factor-alpha (TNFα) is demonstrated to boost the secretion of anti-inflammatory mediators including IL-10 and TGFβ that further participate in immunosuppression and the induction of Tregs [5].

TNFα interacts with two transmembrane receptors with an entirely distinct biological function. TNFα-TNFR1 axis controls the injury and pro-apoptotic pathways while TNFα-TNFR2 mediates protective functions leading to cell proliferation and survival [6–9]. Unlike TNFR1 that is expressed by almost all cells, the TNFR2 expression is limited to few cells such as Tregs, MSCs, neural cells (NCs), regulatory B cells (Bregs), myeloid-derived suppressive cells (MDSCs), and endothelial progenitor cells (EPCs) that are someway involved in immunosuppressive and immunoregulatory utilities [10–13].

Recently, in an attempt to explore the cross-talk between MSCs and T cells, we have demonstrated for the first time that the TNFR2 expression by MSCs is indispensable for their capacity to suppress and decrease the activation phenotype of T cells [11]. Furthermore, hampering the TNFα-TNFR2 signaling pathway in MSCs led to the reduced secretion of IL-10 and TGFβ anti-inflammatory cytokines and enhanced TNFα, INFγ, IL-2, and IL-17 pro-inflammatory cytokines by effector T cells (Teffs) [11]. Interestingly, we demonstrated that compared to MSCs derived from WT mice, their counterparts isolated from TNFR2 KO mice were remarkably less able to convert CD3^+^CD25^−^ Tconvs to CD4^+^Foxp3^+^ Tregs and CD8^+^Foxp3^+^ Tregs [11]. Here in this current article, we have evaluated the impact of TNFR2 expression by T cells and its association with the induction of functional Tregs by MSCs.

## Methods

### MSC isolation and characterization

BM-MSCs were isolated from the femurs and tibias of 4- to 8-week-old C57BL/6 WT mice (Charles River and Envigo) as already described [8, 11]. Cells were cultured in 25 cm^2^ flasks in DMEM (Gibco) containing low glucose, 1% GlutaMAX, 10% FBS, and 1% penicillin/streptomycin-neomycin (P/S/N) (Gibco), hereafter referred to as completed DMEM. Cells were incubated at 37 °C in 5% CO_2_. Non-adherent cells were removed every 8 h; pure MSCs were obtained after 4 to 5 weeks. Cells were sub-cultured prior to confluency. For characterization of MSCs, 10^5^ cells/well were seeded in 96-well round bottom plates and immune-stained with CD44-PE-Vio770, Sca1-APC, CD105-FITC, CD73-PE, CD45-VIOBLUE, CD34-FITC, and CD90-PE (Miltenyi). Unstained cells and isotypes were used as controls. Flow cytometric analysis was performed using LSRFORTESSA flow cytometer (BD Biosciences) and analyzed by FlowJo software v10 (FlowJo LLC).

### T cell isolation

Mouse pan T cell isolation kit (Miltenyi) was used to isolate total CD3^+^ T cells from the spleens of 6- to 12-week-old female WT C57BL/6 mice (Envigo and Charles River) and C57BL/6 TNFR2 KO mice (B6.129S2-Tnfrsf1b^tm1Mwm^/J—The Jackson Laboratory). CD25^+^ cells were depleted from the CD3^+^ T cell population using anti-CD25 biotin-conjugated antibody (BD Biosciences) followed by staining with anti-biotin microbeads (Miltenyi). Cells were then isolated using magnetic-activated cell sorting (MACS). The resulting CD3^+^CD25^−^ WT and TNFR2 KO-T cells (Tconvs) were co-cultured with WT-MSCs.

### Treg induction assay

5 × 10^4^ WT-MSCs were co-cultured in 6-well plates with increasing numbers of mouse WT or TNFR2 KO-Tconvs in a total volume of 2 ml. The ratios of MSCs to T cells were 1:1, 1:2, 1:4, 1:6, 1:8, and 1:10. 10^5^ WT or TNFR2 KO-Tconvs were used as control T cells alone. T cells were stimulated with Dynabeads mouse T-activator CD3/CD28 (Gibco) according to the supplier’s protocol. To eliminate the possibility of a nonspecific effect of media on T cells, they were cultured in 50% RPMI-50% DMEM and compared to culture in 100% RPMI containing 10 % FBS, 1% P/S/N; 1% HEPES and 5x10−5 M β-mercaptoethanol, hereafter referred to as complete RPMI. After 3 days, MSC-exp T cells were harvested and stained using the following Abs: CD4-VIOBLUE, CD8α-FITC, CD25-PE-Vio770 (Miltenyi), and Foxp3-PE-Cy5 (eBioscience).

### MSC-exp T cell suppressive capacity assay

5 × 10^5^ T cells from WT and TNFR2 KO mice were co-cultured with 5 × 10^4^ BM-MSCs WT. After 3 days, total MSC-exp T cells were collected and co-cultured in 96-well round bottom plates with freshly isolated, CFSE-labeled, and CD3/CD28-activated mouse CD3^+^CD25^−^ responder T cells in a fixed 1:10 MSC-exp T cells to responder T cell ratio in complete RPMI medium. 10^5^ CFSE-labeled, activated, and non-activated mouse WT and TNFR2 KO-Tconvs grown alone in culture were used as controls. After 3 days of co-culture, cells were harvested and stained using VIOBLUE-anti-CD4 and PE-Vio770-anti-CD8 antibodies’ combination. The percentage of proliferating cells among CD4^+^ and CD8^+^ T cells was analyzed by flow cytometric measurements of the dilution of CFSE using LSRFORTESSA flow cytometer (BD Biosciences). The T cell proliferation and the division indexes were calculated using an automated analysis feature in FlowJo software v10 (FlowJo LLC).

### ELISA assay

Enzyme-linked immunosorbent assay experiments using Mouse IL-10 Instant ELISA kit and the Mouse TGF beta 1 ELISA kit (Invitrogen, Thermo Fisher Scientific) were performed following the manufacturer’s instructions. Detection of IL-10 and TGFβ was quantified by measuring the absorbance at 450 nm using a plate reader (Multiskan EX, Thermo Fisher Scientific). Results were calculated using linear regression with Excel software (Microsoft Office).

### Statistical analysis

Prism (GraphPad) was used for statistical analysis. The Shapiro-Wilk normality test was performed to assess the normal distribution of data. Then, Student’s *t* test or one-way ANOVA with post hoc analysis was performed depending on the number of comparatives for *P* value generation. Data are the mean value ± SEM. *P < .05, **P < .01, ***P < .001, and ****P < .0001.

## Results

### The expression of TNFR2 by T cells is essential for their conversion into Tregs by MSCs

**Fig. 1:**
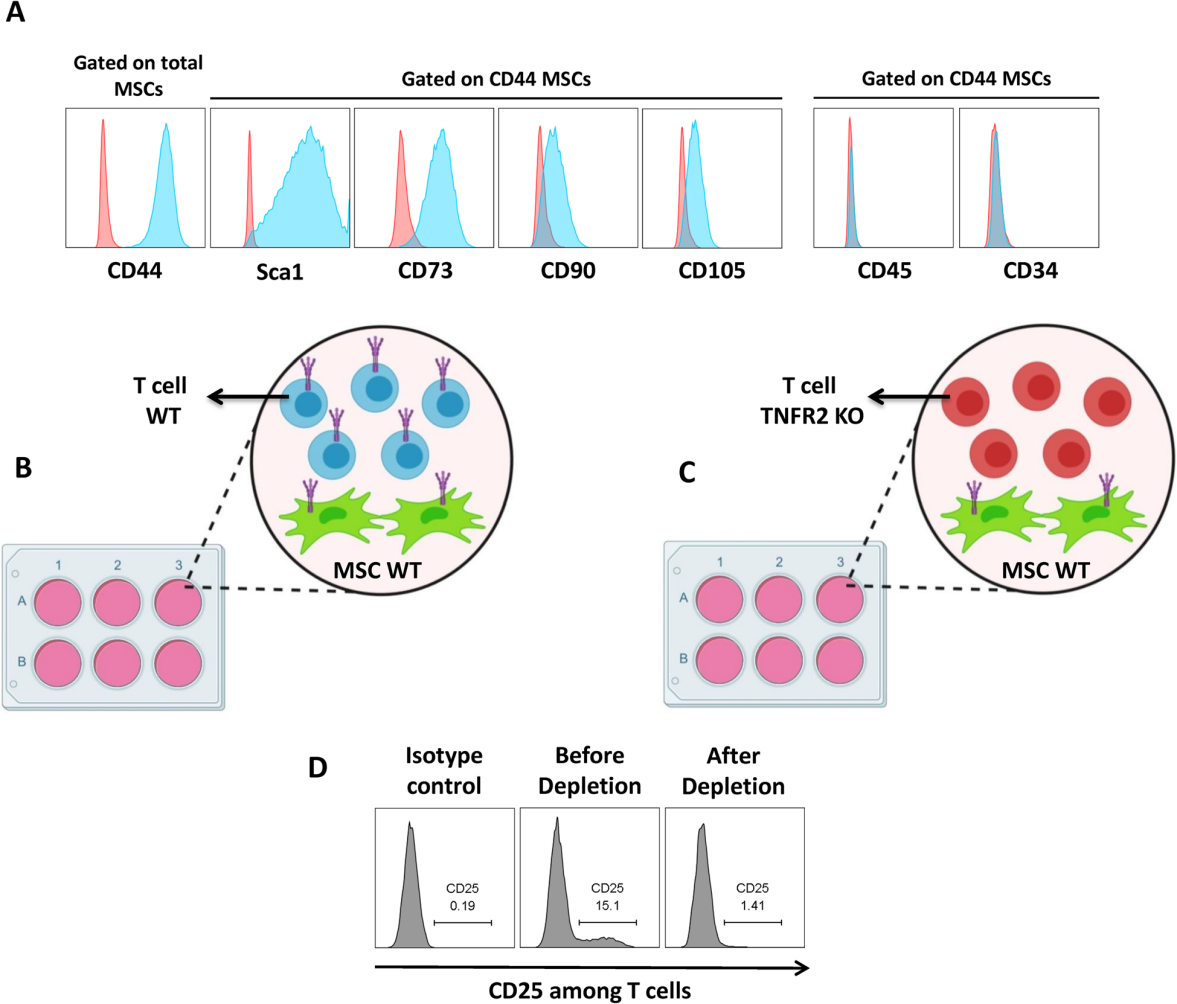
MSC characterization and the schematic representation of the experimental procedures. **a** MSCs isolated from the bone marrow of WT mice were first evaluated for the expression of mouse MSC characterization markers. Cells were gated on total MSCs for evaluation of CD44 marker. For the rest of the markers, cells were first gated on CD44^+^ cells. Red histograms depict the isotype controls, and blue histograms depict the expression of each desired marker. Thereafter, MSCs were co-cultured with **b** WT-T cells and **c** TNFR2 KO-T cells for further experimental procedures. **d** These FACS representatives demonstrate the depletion of CD25^+^ subpopulation from initial T cells

**Fig. 2:**
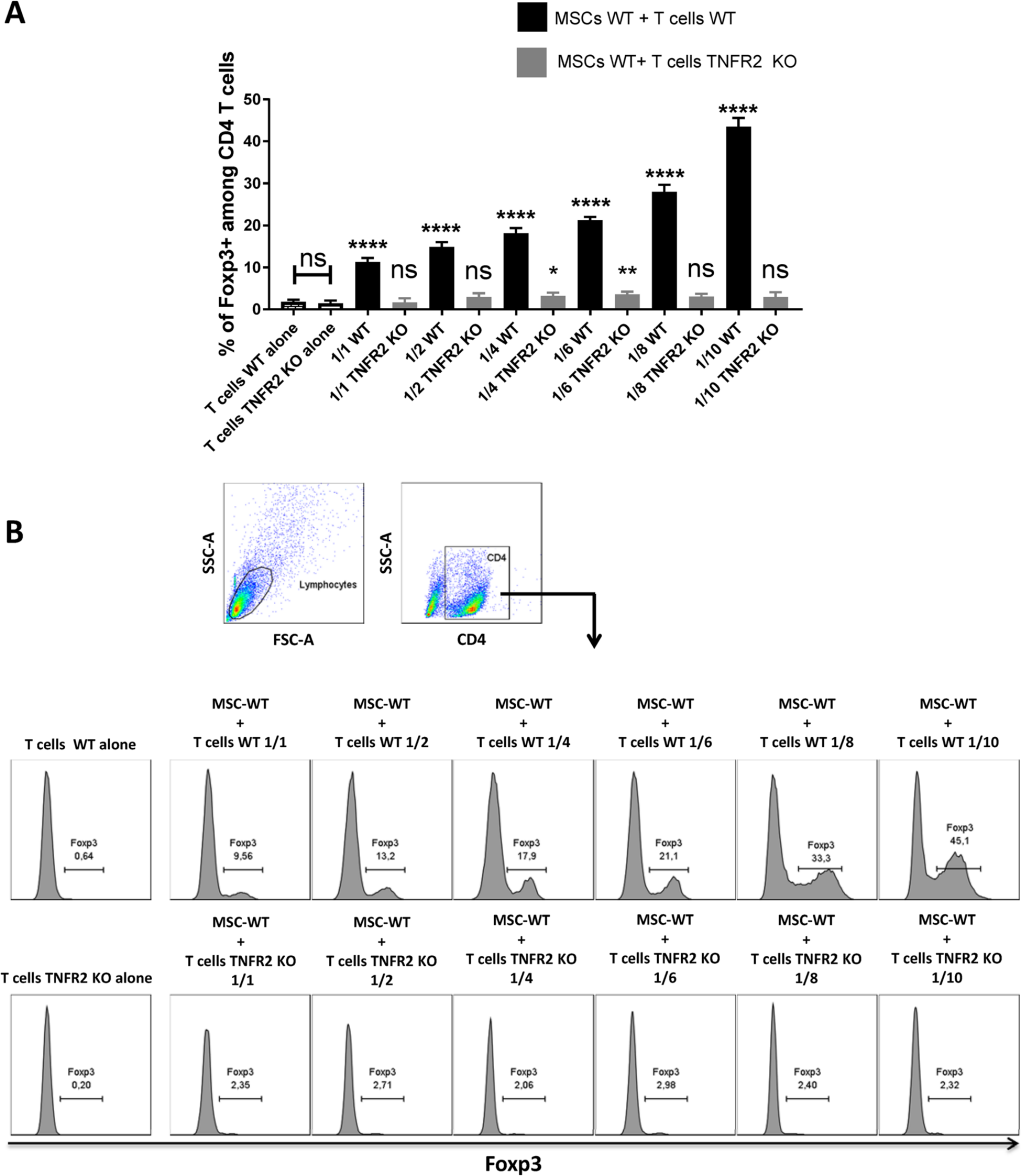
TNFR2 expression by T cells is directly associated with the Foxp3 upregulation by MSCs. MSCs were co-cultured with six increasing doses (1:1 to 1:10) of WT and TNFR2 KO-T cells for a duration of 3 days. **a** Then, they were collected and analyzed by flow cytometry for the expression of the Foxp3 molecule. WT or TNFR2 KO CD3^+^CD25^−^ T cells were used as control T cells alone. The white columns depict WT or TNFR2 KO-T cells cultured alone. The black columns depict WT-T cells co-cultured with WT-MSCs, while gray columns represent TNFR2 KO-T cells co-cultured with WT-MSCs. **b** These FACS plots and histograms demonstrate our T cell gating strategy revealing a dose-dependent upregulation of the Foxp3 transcription factor among WT-T cells. Data are representative of two separate experiments (*n* = 8)

We have first isolated and expanded MSCs from the bone marrow (BM) of C57BL/6 wild type (WT) mice (BM-MSCs WT). The phenotypic characterization confirmed the positive expression of mouse essential MSC markers such as CD44, Sca1, CD73, CD90, and CD105. Furthermore, they did not express CD45 common leukocyte and CD34 hematopoietic markers (Fig. 1a). Thereafter, we have co-cultured 5 × 10^4^ MSCs in passage 3 with six increasing ratios (1:1, 1:2, 1:4, 1:6, 1:8, and 1:10) of mouse CD3^+^CD25^−^ Tconvs freshly isolated from WT C57BL/6 mice (Fig. 1b) or TNFR2 KO (C57BL/6 TNFR2KO-B6.129S2-Tnfrsf1b^tm1Mwm^/J-) mice (Fig. 1c). CD25^+^ subpopulation was eliminated from the initial T cell population (92.40 ± 2.13% of depletion efficiency) in order to deplete the natural CD25^+^Foxp3^+^ Tregs (Fig. 1d). After 3 days, total T cells (cells in suspension) were collected and analyzed by flow cytometry for the expression of the Foxp3 transcription factor which is the key marker of Tregs. Our results revealed that while WT-T cells co-cultured with MSCs had increased Foxp3 expression in a dose-dependent manner, surprisingly, TNFR2 KO-T cells were not converted to Foxp3^+^ induced Tregs (iTregs) (Fig. 2a, b). In this setting, we only observed a slight but significant increase in Foxp3 expression at 1:4 and 1:6 TNFR2 KO-T cell conditions with 3.22% and 3.59% of Foxp3 expression, respectively. Accordingly, in our previous works, we showed that TNFα KO-T cells were less capable of converting towards Foxp3^+^ iTregs, highlighting the importance of TNFα-TNFR2 signaling pathways in Treg induction mechanism by MSCs [11].

### Unlike WT-T cells, TNFR2 KO-T cells co-cultured with MSCs have less anti-inflammatory cytokine production

**Fig. 3:**
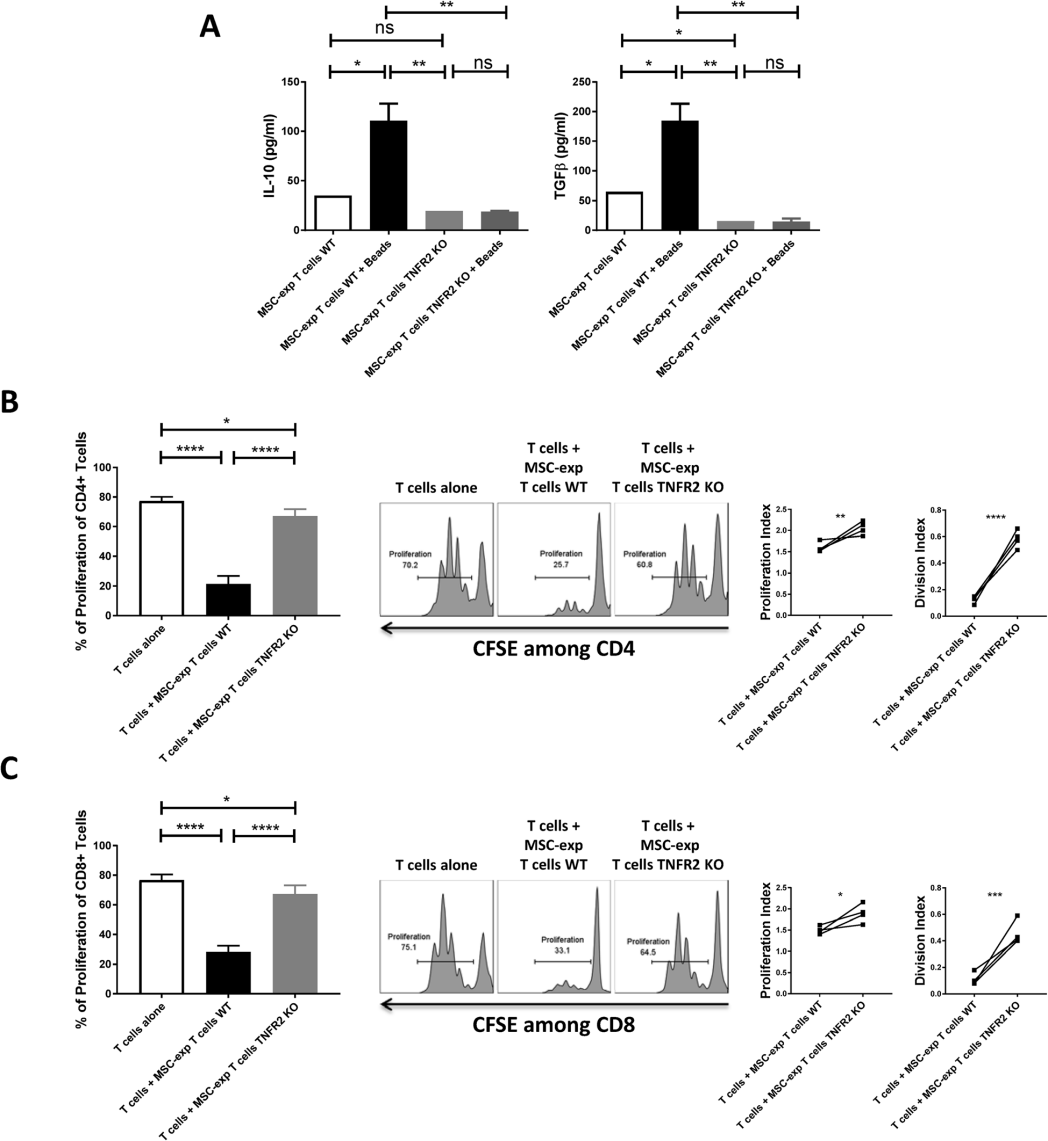
TNFR2 expression by T cells is directly associated with the secretion of anti-inflammatory cytokines and their immunosuppressive effect. **a** The secretion of IL-10 and TGFβ anti-inflammatory cytokines was assessed by the ELISA method. Total T cells collected after co-culturing with WT-MSCs were further activated or not with anti-CD3/CD28 beads for a duration of 24 h. Furthermore, Treg immunosuppressive effect was measured by performing an MLR test. Briefly, 10^4^ MSC-exp T cells were added to 10^5^ freshly isolated CFSE-labeled responder T cells. Responder cells were activated using anti-CD3/CD28 beads. After 3 days, the proliferation capacity of **b** CD4^+^ and **c** CD8^+^ T cell subsets was analyzed by flow cytometry (*n* = 4). FACS histograms demonstrate flow cytometry representatives of the proliferation assay

Although the expression of Foxp3 is the main element in Treg measurement, some studies reported other rare populations of Foxp3-negative Tregs which secrete IL-10 and TGFβ [14]. Therefore, we have further investigated whether WT and TNFR2 KO-T cells co-cultured with WT-MSCs have different IL-10 and TGFβ secretion patterns regardless of their Foxp3 expression. 10^4^ BM-MSCs WT was co-cultured with 10^5^ freshly isolated CD3^+^CD25^−^ Tconvs harvested from WT or TNFR2 KO mice. After 3 days of co-culture, total MSC-exposed T cells (MSC-exp T cells WT and MSC-exp T cells TNFR2 KO) were collected and stimulated or not with anti-CD3/CD28 beads for a duration of 24 h. Interestingly, we have observed an elevated secretion of both cytokines by WT-T cells in comparison to low levels of secretion by TNFR2 KO-T cells (Fig. 3a). Moreover, while activating WT-T cells significantly increased their ability to produce both anti-inflammatory cytokines, there was no difference between non-stimulated and bead-stimulated TNFR2 KO-T cell secretion profile (Fig. 3a).

### Tregs induced from WT-T cells are more suppressive than those induced from TNFR2 KO-T cells

Since we observed some levels of IL-10 and TGFβ secretion even in MSC-exp T cells TNFR2 KO conditions, we sought to assess the immunosuppressive effect of both T cells co-cultured with MSCs. Therefore, 5 × 10^5^ T cells from WT and TNFR2 KO mice were co-cultured with 5 × 10^4^ BM-MSCs WT. After 3 days, total MSC-exp T cells were collected and set in a new MLR test with 10^5^ freshly isolated CFSE-labeled activated CD3^+^CD25^−^ responder T cells in a 1:10 MSC-exp T cell to responder T cell ratio. Three days after, the proliferation of CD4^+^ and CD8^+^ T cells was analyzed. In this setting, measuring the CFSE expression followed by the evaluation of the proliferation and division indexes of T cells demonstrated that Tregs induced from WT-T cells (MSC-exp T cells WT) were significantly more suppressive against both CD4 (Fig. 3b) and CD8 Teffs (Fig. 3c) in comparison to Tregs induced from TNFR2 KO-T cells (MSC-exp T cells TNFR2 KO).

## Discussion and conclusion

Here, we report for the first time a new mechanism in which MSCs and T cells use to interact with each other. Indeed, the expression of TNFR2 by Tregs has been already reported to be crucial for their immunosuppressive effect [15]. Many other research works have previously demonstrated a series of mechanisms that MSCs use to induce Tregs. However, to our knowledge, this is the first evidence for the importance of TNFR2 expression by T cells in this procedure. This work is another evidence for the imperative role of the TNFR2 immune checkpoint molecule in immune regulation. The expression of this molecule is essential by both MSCs and T cells for proper Treg induction. In this study, to evaluate the MSCs’ capacity of Treg induction, we first eliminated CD25^+^Foxp3^+^ natural Tregs by depleting CD25^+^ subpopulation from the initial T cells. Although the depletion process was efficient, the low percentage of remaining CD25^+^ T cells will not exclude the possibility of Treg expansion rather than mere Treg induction by WT-MSCs.

Our data reveal Foxp3 upregulation important but not the only Treg functional marker, at least, in Tregs induced from MSCs. Although MSC-exposed TNFR2 KO-T cells were not able to express Foxp3, they still secreted low levels of IL-10 and TGFβ cytokines leading to some levels of T cell immunosuppression. This makes the TNFR2 expression by T cells one of the most central but not the sole mediator of Treg induction by MSCs.

This work paves the way for further investigations on the association of TNFR2 expression by T cells and the previously described mechanisms of Treg induction by MSCs. Besides, it is another piece of information suggesting the potential implication of anti-TNFR2 therapy for interfering with immunosuppression notably in cancer immunotherapy strategies.

## Data Availability

The datasets used and/or analyzed during the current study are available from the corresponding author on reasonable request.

## Abbreviations

B reg: Regulatory B cell
BM: Bone marrow
EPCs: Endothelial progenitor cells
IL-10: Interleukin-10
Ab: Antibody
MDSCs: Myeloid-derived suppressive cells
MSC-exp T cells: MSC-exposed T cells
MSCs: Mesenchymal stem cells
NCs: Neural cells
Teff: Effector T cell
Tconv: Conventional T cell
Treg: Regulatory T cell
TGFβ: Tumor growth factor-beta
TNFR1: Tumor necrosis factor receptor 1
TNFR2 KO: Tumor necrosis factor receptor 2 knockout
TNFR2: Tumor necrosis factor receptor 2
TNFα: Tumor necrosis factor-alpha
TSDR: Treg-specific *demethylated* region
WT: Wild type

## References

[1] Yu Y, Valderrama AV, Han Z, Uzan G, Naserian S, Oberlin E. Human fetal liver MSCs are more effective than adult bone marrow MSCs for their immunosuppressive, immunomodulatory and Foxp3+ T Regs induction capacity. In Review. 2020. 10.21203/rs.3.rs-40561/v2. 10.1186/s13287-021-02176-1 PMC7888159 33597011

[2] Khosravi M, Bidmeshkipour A, Cohen JL (2018). Induction of CD4+CD25+FOXP3+ regulatory T cells by mesenchymal stem cells is associated with modulation of ubiquitination factors and TSDR demethylation. Stem Cell Res Ther.

[3] Khosravi M, Karimi MH, Hossein Aghdaie M, Kalani M, Naserian S, Bidmeshkipour A (2017). Mesenchymal stem cells can induce regulatory T cells via modulating miR-126a but not miR-10a. Gene..

[4] Khosravi M, Bidmeshkipour A, Moravej A, Hojjat-Assari S, Naserian S, Karimi MH (2018). Induction of CD4+CD25+Foxp3+ regulatory T cells by mesenchymal stem cells is associated with RUNX complex factors. Immunol Res.

[5] Putra A, Ridwan FB, Putridewi AI (2018). The role of TNF-α induced MSCs on suppressive inflammation by increasing TGF-β and IL-10. Open Access Maced J Med Sci.

[6] Barkestani MN, Shamdani S, Bakshloo MA, et al. TNFα priming through its interaction with TNFR2 enhances endothelial progenitor cell immunosuppressive effect: new hope for their widespread clinical application. In Review. 2020. 10.21203/rs.3.rs-71393/v2. 10.1186/s12964-020-00683-x PMC7784277 33397378

[7] Faustman DL, Davis M (2013). TNF receptor 2 and disease: autoimmunity and regenerative medicine. Front Immunol.

[8] Beldi G, Bahiraii S, Lezin C, et al. TNFR2 is a crucial hub controlling mesenchymal stem cell biological and functional properties. Front Cell Dev Biol. 2020;8. 10.3389/fcell.2020.596831. 10.3389/fcell.2020.596831 PMC7746825 33344453

[9] Naserian S, Shamdani S, Uzan G. Current preventions and treatments of aGVHD: from pharmacological prophylaxis to innovative therapies. Front Immunol. 2020;11. 10.3389/fimmu.2020.607030. 10.3389/fimmu.2020.607030 PMC7773902 33391276

[10] Naserian S, Abdelgawad ME, Afshar Bakshloo M (2020). The TNF/TNFR2 signaling pathway is a key regulatory factor in endothelial progenitor cell immunosuppressive effect. Cell Commun Signal CCS.

[11] Beldi G, Khosravi M, Abdelgawad ME (2020). TNFα/TNFR2 signaling pathway: an active immune checkpoint for mesenchymal stem cell immunoregulatory function. Stem Cell Res Ther.

[12] Shamdani S, Uzan G, Naserian S (2020). TNFα-TNFR2 signaling pathway in control of the neural stem/progenitor cell immunosuppressive effect: different experimental approaches to assess this hypothetical mechanism behind their immunological function. Stem Cell Res Ther.

[13] Chen Z, Palmer TD (2013). Differential roles of TNFR1 and TNFR2 signaling in adult hippocampal neurogenesis. Brain Behav Immun.

[14] Ballke C, Gran E, Baekkevold ES, Jahnsen FL (2016). Characterization of regulatory T-cell markers in CD4+ T cells of the upper airway mucosa. PLoS One.

[15] Leclerc M, Naserian S, Pilon C (2016). Control of GVHD by regulatory T cells depends on TNF produced by T cells and TNFR2 expressed by regulatory T cells. Blood..

